# Phase-Field Modeling of Freeze Concentration of Protein Solutions

**DOI:** 10.3390/polym11010010

**Published:** 2018-12-21

**Authors:** Tai-Hsi Fan, Ji-Qin Li, Bruna Minatovicz, Elizabeth Soha, Li Sun, Sajal Patel, Bodhisattwa Chaudhuri, Robin Bogner

**Affiliations:** 1 Department of Mechanical Engineering, University of Connecticut, Storrs, CT 06269, USA; jiqin.li@uconn.edu (J.-Q.L.); elizabeth.soha@uconn.edu (E.S.); 2 School of Pharmacy, University of Connecticut, Storrs, CT 06269, USA; bruna.minatovicz@uconn.edu (B.M.); bodhi.chaudhuri@uconn.edu (B.C.); robin.bogner@uconn.edu (R.B.); 3 Regeneron Pharmaceuticals Inc., Tarrytown, NY 10591, USA; li.sun@regeneron.com; 4 MedImmune, Gaithersburg, MD 20878, USA; patelsaj@medimmune.com; 5 Institute of Materials Science, University of Connecticut, Storrs, CT 06269, USA

**Keywords:** phase-field modeling, freeze concentration, space confinement, freezing of biologics, ice crystals, supercooled water

## Abstract

Bulk solutions of therapeutic proteins are often frozen for long-term storage. During the freezing process, proteins in liquid solution redistribute and segregate in the interstitial space between ice crystals. This is due to solute exclusion from ice crystals, higher viscosity of the concentrated solution, and space confinement between crystals. Such segregation may have a negative impact on the native conformation of protein molecules. To better understand the mechanisms, we developed a phase-field model to describe the growth of ice crystals and the dynamics of freeze concentration at the mesoscale based on mean field approximation of solute concentration and the underlying heat, mass and momentum transport phenomena. The model focuses on evolution of the interfaces between liquid solution and ice crystals, and the degree of solute concentration due to partition, diffusive, and convective effects. The growth of crystals is driven by cooling of the bulk solution, but suppressed by a higher solute concentration due to increase of solution viscosity, decrease of freezing point, and the release of latent heat. The results demonstrate the interplay of solute exclusion, space confinement, heat transfer, coalescence of crystals, and the dynamic formation of narrow gaps between crystals and Plateau border areas along with correlations of thermophysical properties in the supercooled regime.

## 1. Introduction

Understanding the mechanisms and process impacts of freezing on protein solutions is critical in the pharmaceutical industry for the manufacturing of high quality biologics. During the freezing process, the heat, mass, and momentum transport influence the size, shape, and growth rate of ice crystals as well as the distribution of protein molecules in between. When a frozen product is freeze-dried, the ice crystal size and distribution resulting from freezing determine the sublimation kinetics during primary drying, and eventually the stability of the biologics [1,2,3,4]. Freeze concentration occurs due to the exclusion of protein and other solutes from ice crystals and space confinement between ice/freeze concentrate interfaces. Overall, the seeding or nucleation pattern and the freezing rate control the growth of ice crystals. Current understanding is that at fast cooling rates, dendritic crystals branch out quickly, trapping proteins among side branches of crystals to yield a more uniform protein distribution. In contrast, at slow cooling rates, protein molecules redistribute while cellular (instead of dendritic) crystal structure forms, resulting in a more significant concentration polarization as crystals continue to grow and coalesce. Freeze concentration and its impact on protein stability is more prominent during large scale freezing. Although rapid freezing is likely advantageous because of more uniform protein distribution in the frozen product, it can be difficult to achieve at the manufacturing scale. Experimentally it has been observed that upon freezing the local thermal and convective effects induced by phase transition, cooling, gravity, control of ice nucleation for freeze-drying, and protein residence time in the freeze concentrates all have impacts on the process performance and the distribution and stability of proteins [5,6,7,8].

Qualitative and quantitative analyses of the relevant dynamics in freeze concentration are complicated by the coupling of thermal and multi-component mass transport, phase transition physics, protein interactions with co-solutes, morphological change and the space confinement effect, thermophysical properties at the supercooled regime, and molecular crowding and protein-protein interactions. Although casting in conventional thermal manufacturing process is a well-established technology, where the relevant physics such as dendritic growth, patern formation, and microsegregation of pure and alloy systems has been studied extensively [9,10,11,12,13,14,15], the existing theoretical models and experimental results are not readily applicable to describe freezing of protein biologics. Even at the laboratory scale, only a few investigations on the phase transition of protein solutions have focused on fundamental heat transfer, phase change, and computational fluid dynamics simulations. Nakagawa et al. [16] proposed a thermal conduction model using the classical enthalpy method to trace phase change and temperature distribution during freezing of mannitol and BSA based solutions in a vial. Without further description of the growth dynamics of ice crystals, the estimated sizes of crystals and the freeze-dried layer permeability correlate well with the freezing front rate and temperature gradient in the frozen zone. Radmanovic et al. [17] modeled the heat transfer involved in freezing of monoclonal antibody solution and evaluated the product quality based on the size and polydispersity of the protein aggregates. Roessl et al. applied volume-of-fluid computational fluid dynamics to simulate the freezing process and compared the results with measurements of temperature and cryoconcentration fields [18]. On a smaller scale, Butler observed the solute profile at the ice/freeze concentrate interface due to freeze concentration using optical interferometry [19]. Kaempfer and Plapp developed phase-field modeling of sublimation dynamics and relevant vapor and thermal transport on the microstructure of dry snow and compared with microtomography images [20]. van der Sman developed a phase-field model to simulate growth and suppression of ice crystals in sugar solutions [21], and successfully incorporated polymer mean field theory into the phase transition dynamics with simplified thermal effect. Huang et al. [22] revealed directional growth of anisotropic ice crystals in ceramic colloidal suspensions, with the resulting porous material having potential for biomedical applications such as functional materials for implants. These relevant studies have only focused on either thermal or composition with phase transition in the freezing process, however, at the mesoscale in particular, multiple physics are often convoluted in the phase transition dynamics, requiring the inclusion of the effects of thermal, fluid flow, thermal mechanical response, composition and changes of thermophysical properties into the analysis [23,24]. Here we propose a new model to integrate these effects and provide mathematical descriptions and simulation of the freeze concentration effect.

The phase-field method is an Eulerian approach that naturally resolves fusion, merging, splitting, topological and morphological changes of dynamic interfaces and thermodynamic states of the materials involved. The underlying concept of a smooth transition between phases and the contribution of non-local or gradient energy was initially introduced by van der Waals in 1893 [25] to describe liquid-vapor phase transition over a diffuse interface. A general phase transition process is driven by the increase of entropy or reduction of free energy. The gradient effect due to spatial variation of the phase-field function or order parameter eventually leads to a continuous phase-field equation that describes the local state of the material, and can clearly distinguish the location of the transition interface between phases. This approach was further developed in many research areas in physics and material sciences, known as Ginzburg-Landau free energy theory to describe superconductivity, Allen–Cahn model to descibe transition dynamics of non-conserved phase field or order parameter, Cahn–Hilliard model for conserved parameters such as density or concentration, and Model A/B for isothermal and H for nonisothermal fluids near a critical state [26,27]. The phase-field function is uniform within a homogeneous phase, but has a narrow and smooth transition profile across the interface between phases. Rigorous derivation of phase-field equations for non-isothermal systems is based on the principles of irreversible thermodynamics [28,29], in which the entropy functional of the system, entropy transport equation, and the 2nd law of thermodynamics are the starting point of derivation. The phase-field method has been widely applied to model dendritic pattern formation in material sciences since the works of Kobayashi [9], Warren and Boettinger [10], Murray, Wheeler, and Glicksman [11], and Karma and Rappel [12,13] in the 1990s. The transient evolution of the solidification interfaces is driven by thermal gradient, interfacial instability, Gibbs–Thompson kinetics, and anisotropic interfacial energy along with hydrodynamics effect, which all play an important role in pattern formation, solute distribution, and coarsening and remelting of dendritic microstructures [14,15,30,31]. The method has been further extended to address multi-component problems with and without convective effect, and for a variety of applications in thermal fluid sciences and applied mechanics including geohydraulics, fracture mechanics, multiphase flows, and reaction engineering etc. [32,33,34,35]. In this paper we apply phase-field method to model freeze concentration of protein solutions and observe concentration polarization without considering anisotropic dendritic formation. Instead of anisotropic interfacial energy, the focus here is to couple the evolving dynamics of crystals with fluid flow, heat transfer, protein diffusion, and the convective transport induced by gravity effect and density variation across the moving ice/freeze-concentrate interface. In the mesoscale domain, the cooling effect is simplified by using a representative bulk cooling rate, which reduces the computational cost and potentially can be extended and integrated with multiscale analysis including container configuration and experimental settings at a larger scale.

## 2. Theoretical Analysis

**Figure 1:**
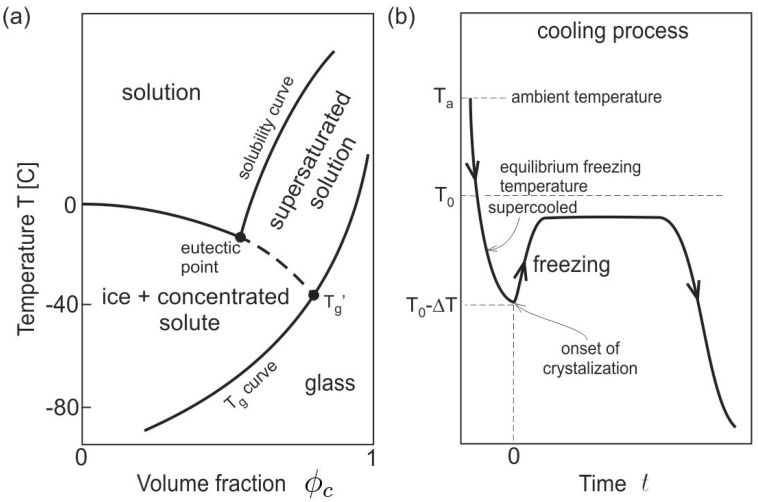
(**a**) Temperature–composition phase diagram of a binary protein solution including freezing, solubility, glass transition curves, and material states. (**b**) Schematic of the cooling process in terms of the average temperature of the representative volume versus time, and with initial condition defined at the crystallization point with temperature $T_{0}-\Delta T$, where the subcooled temperature ΔT is used for temperature scaling.

A challenge in the theoretical analysis is to compile relevant thermophysical properties of protein solutions as in crystal or supercooled liquid state, including density, specific heat, thermal conductivity, mass diffusivity, and dynamic viscosity. These physicochemical properties in general depend on temperature and solute concentration, and the available measurements are very limited. Here we consider sucrose solution to demonstrate the modeling results based on available data. Although sucrose has very different properties than proteins, it is a very common excipient used as a cryoprotectant in protein formulations, freeze concentration of sucrose may also impact protein stability, and thus the investigation is very relevant to the biopharmaceutical industry. The temperature–composition phase diagram of a binary model protein solution (e.g., sucrose and water) at ambient pressure is shown in Figure 1a [6,36]. The freezing curve (liquidus) shows freezing point depression, and the extension of the freezing curve intercepts the glass transition curve at the ${T_{g}}^{\prime }$ point, where the solute concentration reaches the maximum value. Freezing of protein solutions often starts from room temperature to a supercooled liquid state (Figure 1b) with a few to tens of degree Kelvin below the equilibrium freezing temperature at a given composition. The temperature raises due to heat release from freezing or solidification, possibly near the equilibrium freezing temperature and eventually drops due to the cooling rate applied to the material volume. In this study, assuming that the heterogeneous nucleation can be controlled by random seeding, ultrasound, electrical, or other freezing methods [3], the supercooled temperature and the size and locations of seeding are predetermined as the initial conditions, and then the simulation proceeds as a non-equilibrium freezing process. The continuous increase in viscosity and change of local freezing point from freeze concentration are considered. The following assumptions are made to facilitate and simplify the theoretical analysis and computation: (i) ice/freeze concentrate interfacial energy is assumed half of the free surface energy of water, (ii) the interfacial energy is assumed isotropic or no preferential directions for the formation of dendritic pattern, which can be considered in further investigations, (iii) thermal expansion, thermal stress, or elasticity in the crystal phase are neglected, and in fact ice crystals are treated as a highly viscous fluid with dynamic viscosity at least four orders of magnitude larger than fluid, (iv) entanglement or crystallization of the solute molecules at high concentrations is not considered as the corresponding mean field theory is much more complicated, (v) adsorption and molecular interactions of proteins with the ice/freeze concentrate interfaces are not considered, (vi) thermal radiation and van der Waals force between nearby interfaces may have effects on heat transfer and interfacial dynamics at the mesoscale, but are assumed negligible in this work.

### 2.1. Thermodynamics Approach

Irreversible thermodynamics provides a rigorous route to derive the phase-field governing equations for a non-isothermal system involved in thermal manufactuing processes, in which the coupling of phase-field functions with other transport equations is important. Here we consider phase field $\phi_{s\ell }\left(r,t\right)$ as a function of location r and time *t* to distinguish the solid (ice crystal, $\phi_{s\ell }=1$) and liquid (liquid protein solution, $\phi_{s\ell }=-1$) phases, and the ice/freeze concentrate interface has a narrrow but smooth transition of $\phi_{s\ell }$ between −1 to 1. The 2nd phase-field function is the protein volume fraction $\phi_{c}\left(r,t\right)$ as a conserved parameter at the range of 0–1. To derive the thermodynamically consistent formulation we follow the approach proposed by Penrose and Fife [28] and Wang et al. [29]. Starting from the entropy functional of the material volume that takes the contributions of the entropy density in the bulk phase and the non-local or gradient effects due to spatial variation of the phase fields $\phi_{s\ell }$ and $\phi_{c}$ into account, we have

(1) $$S=\int_{\Omega }\left[\rho s\left(e,\phi_{s\ell },\phi_{c}\right)-\frac{1}{2}\xi_{s\ell }^{2}|\nabla \phi_{s\ell }{|}^{2}-\frac{1}{2}\xi_{c}^{2}{\left|\nabla \phi_{c}\right|}^{2}\right]dV,$$

where ρ is density, *s* is specific entropy as a function of specific internal energy *e* and the two phase-field functions, and $\xi_{s\ell }$ and $\xi_{c}$ are the assumed constant coefficients corresponding to the gradient effects [15,29]. The gradient coefficient $\xi_{s\ell }$ is connected with reference density $\rho_{0}$, solid-liquid energy barrier coefficient $h_{s\ell }$, and the characteristic interfacial thickness $W_{s\ell }$ as

(2) $$\xi_{s\ell }^{2}=\rho_{0}h_{s\ell }W_{s\ell }^{2}.$$

The solid-liquid interfacial energy $\gamma_{s\ell }$ or the equivalent surface excess energy across the smooth interface is further associated with the above parameters through a one-dimensional approximation, expressed as

(3) $$\gamma_{s\ell }≃\rho_{0}h_{s\ell }W_{s\ell }^{2}T_{0}\int_{-\infty }^{\infty }{\left(\frac{d\phi_{s\ell }}{dx}\right)}^{2}dx=\frac{2\sqrt{2}}{3}\rho_{0}h_{s\ell }W_{s\ell }T_{0},$$

where the factor $2\sqrt{2}/3$ comes from the hyperbolic tangent function to approximate $\phi_{s\ell }$ in equilibrium, which has a defined value from −1 to 1, and the reference temperature $T_{0}$ here represents the equilibrium freezing temperature of pure water. We assume $\gamma_{s\ell }≃\left(1/2\right)\gamma_{g}$ at the ice/freeze concentrate interface, where $\gamma_{g}$ is the surface tension of the free surface of water at $T_{0}$, and all intensive thermodynamic properties are defined as per unit mass quantity.

By applying the Reynolds transport theorem to the entropy of the system, the general differential entropy transport equation can be written as

(4) $$\begin{array}{c} \rho \frac{Ds}{Dt}-\frac{1}{2}\xi_{s\ell }^{2}\frac{D}{Dt}|\nabla \phi_{s\ell }{|}^{2}-\frac{1}{2}\xi_{c}^{2}\frac{D}{Dt}{\left|\nabla \phi_{c}\right|}^{2}=-\nabla \cdot J_{s}+\dot{\Gamma }-\frac{\dot{\Omega }}{T}, \end{array}$$

where *t* is time, D/Dt≡∂/∂t+v·∇ indicates material derivative, $J_{s}$ represents entropy flux, $\dot{\Gamma }$ is the rate of entropy production, and $\dot{\Omega }$ is a heat sink to mimic the cooling from the bulk fluid flow around the mesoscale volume. Considering internal energy $e=e(s,\phi_{s\ell },\phi_{c})$, $de=Tds+\left(\partial e/\partial \phi_{s\ell }\right)d\phi_{s\ell }+\left(\partial e/\partial \phi_{c}\right)d\phi_{c}$, and thus $ds=de/T-\left(\partial e/\partial \phi_{s\ell }\right)d\phi_{s\ell }/T-\left(\partial e/\partial \phi_{c}\right)d\phi_{c}/T$, the time rate of change of entropy Ds/Dt in the transport equation (Equation (4)) can be replaced by the material derivatives of internal energy and the two phase fields, expressed as

(5) $$\begin{array}{c} \frac{Ds}{Dt}=\frac{1}{T}\frac{De}{Dt}-\frac{1}{T}\frac{\partial e}{\partial \phi_{s\ell }}\frac{D\phi_{s\ell }}{Dt}-\frac{1}{T}\frac{\partial e}{\partial \phi_{c}}\frac{D\phi_{c}}{Dt}, \end{array}$$

where the three material derivatives on the right-hand side eventually lead to the thermal energy equation and two phase-field equations. Substituting Equation (5) into (4) and by separating the contributions of entropy flux and entropy production terms due to heat conduction and the evolution of phase fields, that is,

(6) $$\begin{array}{c} \frac{\rho }{T}\frac{De}{Dt}=-\frac{1}{T}\nabla \cdot \dot{q}-\frac{\dot{\Omega }}{T}=-\nabla \cdot \left(\frac{\dot{q}}{T}\right)+\dot{q}\cdot \nabla \left(\frac{1}{T}\right)-\frac{\dot{\Omega }}{T}, \end{array}$$

(7) $$\begin{array}{c} \frac{1}{2}\frac{D}{Dt}{\left|\nabla \phi_{s\ell }\right|}^{2}=\nabla \cdot \left(\nabla \phi_{s\ell }\frac{D\phi_{s\ell }}{Dt}\right)-\frac{D\phi_{s\ell }}{Dt}\nabla^{2}\phi_{s\ell }, \end{array}$$

and

(8) $$\begin{array}{c} \frac{1}{2}\frac{D}{Dt}{\left|\nabla \phi_{c}\right|}^{2}=\nabla \cdot \left(\nabla \phi_{c}\frac{D\phi_{c}}{Dt}\right)-\frac{D\phi_{c}}{Dt}\nabla^{2}\phi_{c}, \end{array}$$

where $\dot{q}=-k_{T}\nabla T$ is the conduction heat flux and $k_{T}$ is the thermal conductivity. The resulting positive entropy production rate can be obtained and expressed as

(9) $$\begin{array}{c} \dot{\Gamma }=\dot{q}\cdot \nabla \left(\frac{1}{T}\right)+\left[\xi_{s\ell }^{2}\nabla^{2}\phi_{s\ell }-\frac{\rho }{T}\frac{\partial e}{\partial \phi_{s\ell }}\right]\frac{D\phi_{s\ell }}{Dt}+\left[\xi_{c}^{2}\nabla^{2}\phi_{c}-\frac{\rho }{T}\frac{\partial e}{\partial \phi_{c}}\right]\frac{D\phi_{c}}{Dt}\geq 0, \end{array}$$

in which the viscous dissipation and capillary work have been neglected due to relatively low dissipated energy compared to heat conduction (small Brinkman number). To accommodate the positive entropy production, one can conclude the non-conserved phase-field equation for the growth of ice crystals as

(10) $$\begin{array}{c} \frac{D\phi_{s\ell }}{Dt}=M_{s\ell }\left[\xi_{s\ell }^{2}\nabla^{2}\phi_{s\ell }-\frac{\rho }{T}\frac{\partial e}{\partial \phi_{s\ell }}\right], \end{array}$$

and the conserved phase-field equation for tracking the single-component protein volume fraction:

(11) $$\begin{array}{c} \frac{D\phi_{c}}{Dt}=-\nabla \cdot J_{\phi_{c}}=-\nabla \cdot \left[M_{c}\left(\phi_{s\ell },\phi_{c}\right)\nabla \frac{\delta S}{\delta \phi_{c}}\right]=\nabla \cdot \left[M_{c}\nabla \left(\frac{\rho }{T}\frac{\partial e}{\partial \phi_{c}}-\xi_{c}^{2}\nabla^{2}\phi_{c}\right)\right], \end{array}$$

where $J_{\phi_{c}}$ is the species flux, $\delta S/\delta \phi_{c}$ is the first variation of the entropy functional, and $M_{s\ell }$ and $M_{c}$ are assumed positive interfacial mobility coefficients. Equations (10) and (11) are essentially the Allen–Cahn (2nd-order) and Cahn–Hilliard (4th-order) equations for a non-isothermal system, respectively. The mobility $M_{s\ell }$ may be acquired empirically or from scaling and asymptotic estimation of the the evolution kinetics of the diffuse interface [12], whereas $M_{c}$ is associated with the classical Fickian diffusion coefficient.

### 2.2. Internal Energy and Free Energy

The specific internal energy including solid and liquid phases and mixing energy of proteins in solutions can be approximated by

(12) $$\begin{array}{c} e\left(T,\phi_{s\ell },\phi_{c}\right)=e_{s\ell }\left(T,\phi_{s\ell }\right)+RT\chi \left(\phi_{s\ell }\right)G\left(\phi_{c}\right), \end{array}$$

where $e_{s\ell }$ represents the internal energy of solid to liquid phases, *R* is gas constant, *T* is temperature, and χ is the Flory’s interaction parameter of regular solutions, and along with the *G* function the mixing term indicates the increase of internal energy due to mixing of water and proteins in either liquid or crystal phases. Here we assume χ>0 (net repulsion between interacting species). The typical *G* function for the mixing effect can be replaced by a double-well potential for keeping $\phi_{c}$ within the range of 0 to 1 in the phase-field computation, written as

(13) $$\begin{array}{c} G\left(\phi_{c}\right)=\phi_{c}\left(1-\phi_{c}\right)∼4\phi_{c}^{2}{\left(1-\phi_{c}\right)}^{2}. \end{array}$$

Furthermore, the internal energy $e_{s\ell }$ can be formulated as

(14) $$\begin{array}{c} e_{s\ell }\left(T,\phi_{s\ell }\right)=e_{s}\left(T\right)+P\left(\phi_{s\ell }\right)L_{a}=e_{\ell }\left(T\right)+\left[P\left(\phi_{s\ell }\right)-1\right]L_{a}, \end{array}$$

where the subscript *s* and *ℓ* indicate homogeneous solid and liquid phases, respectively, and $L_{a}$ is the assumed constant latent heat based on the reference freezing temperature $T_{0}$ of pure water at equilibrium. Developed by Wang et al. [29], the 5th-order interpolation polynomial $P(\phi_{s\ell })$ describes a smooth transition of the internal energy between the liquid ($P=1,\phi_{s\ell }=-1$) and solid ($P=0,\phi_{s\ell }=1$) phases. Here a *P* function that satisfies $P^{\prime }=P^{\prime \prime }=0$ at $\phi_{s\ell }=\pm 1$ is

(15) $$\begin{array}{c} P\left(\phi_{s\ell }\right)=\frac{1}{2}-\frac{15}{16}\left(\frac{1}{5}\phi_{s\ell }^{5}-\frac{2}{3}\phi_{s\ell }^{3}+\phi_{s\ell }\right), \end{array}$$

which gives P(1)=0 and P(−1)=1 so that $e_{s\ell }\left(T,1\right)=e_{s}\left(T\right)$ and $e_{s\ell }\left(T,-1\right)=e_{\ell }\left(T\right)$. The *P* function can be widely used for other thermodynamic properties for a smooth transition between solid and liquid phases. For example, we define Flory’s parameter as

(16) $$\begin{array}{c} \chi \left(\phi_{s\ell }\right)=P\chi_{\ell }+\left(1-P\right)\chi_{s}, \end{array}$$

with the interaction parameters $\chi_{s}>\chi_{\ell }$, which adjusts the partition or exclusion effect for the proteins at the freezing front. Proteins are mostly excluded from ice crystals due to relatively high mixing energy, and are soluble (within the solubility limit) in the liquid phase (Figure 1a).

Similar to the model proposed for binary alloy systems [15,32], in a regular protein solution the free energy density can be obtained by superposing the contributions of pure solid and liquid water, proteins, and the mixing entropy and enthalpy effects based on Flory-Huggins mean field theory for polymer solutions [21,37], applicable for polymer solutions with temperature above $T_{g}$. Here we incorporate the *G* function into to the free energy as

(17) $$\begin{array}{c} f\left(T,\phi_{s\ell },\phi_{c}\right)=\left(1-\phi_{c}\right)f_{s\ell }+\phi_{c}f_{c}\\ \;\;\;+RT\left[\frac{1}{N}\phi_{c}\ln\left(\phi_{c}\right)+\left(1-\phi_{c}\right)\ln\left(1-\phi_{c}\right)+\chi \left(\phi_{s\ell }\right)G\left(\phi_{c}\right)\right], \end{array}$$

where *N* accommodates the size effect as the protein-to-water ratio of partial molar volume, and the Flory’s χ parameter, assumed independent of solute volume fraction, controls the energy barrier in the mixing enthalpy term to ensure exclusion of proteins from the ice crystals. The free energy of ice and liquid water $f_{s\ell }$ drives the phase transition based on the difference of local temperature to the equlibrium freezing point $T_{eq}$, which follows the Gibbs–Thompson effect. Here we assume the interfacial curvature effect is negligible in the mesoscale model, and the equilibrium freezing temperature, $T_{eq}=T_{eq}\left(\phi_{c}\right)$, follows the liquidus line. From the Gibbs–Helmholtz relation, the free energy that describes the latent heat effect for the solid-liquid phase transition dynamics [29] can be expressed as

(18) $$\begin{array}{c} f_{s\ell }\left(T,\phi_{s\ell }\right)=-T\int_{T_{eq}}^{T}\frac{e_{s\ell }}{T^{\prime 2}}dT^{\prime }+\frac{Tf_{s\ell }\left(T_{eq},\phi_{s\ell }\right)}{T_{eq}}, \end{array}$$

where the first term is the driving force for freezing as temperature is different from the reference point $T_{eq}$, whereas the 2nd term is often described by a double-well potential with free energy minima for the the ice or liquid phases at $T=T_{eq}$, written as

(19) $$f_{s\ell }\left(T_{eq},\phi_{s\ell }\right)=\frac{1}{4}h_{s\ell }T_{eq}{\left(1-\phi_{s\ell }^{2}\right)}^{2},$$

where $h_{s\ell }$ is the energy barrier coefficient for the phase transition. The free energy has two minima at $\phi_{s\ell }=-1$ (liquid) and $\phi_{s\ell }=1$ (solid) at the equilibrium freezing temperature $T_{eq}$.

From the internal energy and free energy above, the derivative of internal energy in Equation (10) thus can be expanded and described by the following expression:

(20) $$\begin{array}{cc} \frac{\partial e}{\partial \phi_{s\ell }}{)}_{s,\phi_{c}}=\frac{\partial f}{\partial \phi_{s\ell }}{)}_{T,\phi_{c}}= & \left(1-\phi_{c}\right)\left[-P^{\prime }L_{a}\frac{T-T_{eq}}{T_{eq}}-Th_{s\ell }\left(\phi_{s\ell }-\phi_{s\ell }^{3}\right)\right]\\ & +RT\chi^{\prime }\left(\phi_{s\ell }\right)G\left(\phi_{c}\right). \end{array}$$

Therefore, Equation (10) becomes

(21) $$\begin{array}{c} \frac{D\phi_{s\ell }}{Dt}=M_{s\ell }\left\{\xi_{s\ell }^{2}\nabla^{2}\phi_{s\ell }+\left(1-\phi_{c}\right)\left[\rho P^{\prime }L_{a}\frac{T-T_{eq}}{TT_{eq}}+\rho h_{sl}\left(\phi_{s\ell }-\phi_{s\ell }^{3}\right)\right]-\rho R\chi^{\prime }G\right\}, \end{array}$$

where the transient evolution of the phase field $\phi_{s\ell }$ is now determined by the balance of diffusion, latent heat, double-well potential, and the effect of freezing point depression at higher protein volume fractions. The diffusion effect tends to smear out the interface, the double-well potential keeps the bulk phases separated, whereas the temperature difference along with the latent heat effect provides a driving force for freezing. The increase of protein concentration at the interface suppresses (slows down) the growth of ice crystals due to freezing point depression and the increase of local viscosity at higher solute concentration. Similarly, the derivative of internal energy in Equation (11) can be expanded as

(22) $$\begin{array}{c} \frac{\partial e}{\partial \phi_{c}}{)}_{s,\phi_{s\ell }}=\frac{\partial f}{\partial \phi_{c}}{)}_{T,\phi_{s\ell }}≃RT\left[\frac{1}{N}\left(1+\ln\phi_{c}\right)-\ln\left(1-\phi_{c}\right)-1+\chi G^{\prime }\right], \end{array}$$

where the free energy difference between the ground states $f_{c}$ and $f_{s\ell }$ has been neglected. Therefore the phase-field Equation (11) that governs the transient distribution of protein volume fraction reduces to

(23) $$\begin{array}{c} \frac{D\phi_{c}}{Dt}=\nabla \cdot \left\{M_{c}\nabla \left[\rho R\left(\frac{\ln\phi_{c}}{N}-\ln\left(1-\phi_{c}\right)+\chi G^{\prime }\right)-\xi_{c}^{2}\nabla^{2}\phi_{c}\right]\right\}. \end{array}$$

The above Cahn–Hilliard type model recovers to a conserved, Fickian diffusion equation by defining the mobility as

(24) $$\begin{array}{c} M_{c}=\frac{D(\phi_{c},\phi_{s\ell },T)}{\rho R}\phi_{c}\left(1-\phi_{c}\right). \end{array}$$

The diffusivity *D* in liquid and crystal phases can be scaled (represented by tilde over a variable) and interpolated as

(25) $$\begin{array}{c} D\left(\phi_{s\ell },\phi_{c},T\right)=D_{0}\tilde{D}≃D_{0}\left[P\left(\phi_{s\ell }\right){\tilde{D}}_{\ell }\left(\phi_{c},T\right)+\left(1-P\right){\tilde{D}}_{s}\right], \end{array}$$

where ${\tilde{D}}_{\ell }$ is a concentration- and temperature-dependent protein diffusivity in liquid solution and ${\tilde{D}}_{s}$ is a constant diffusivity in the crystal phase, and both are scaled by reference value $D_{0}$. The subscripts *s* and *ℓ* hereafter denote the solid and liquid phases, respectively. We assume ${\tilde{D}}_{s}\ll {\tilde{D}}_{\ell }$ and the reference diffusivity $D_{0}=D_{\ell }\left(\phi_{c}\to 0,\;T=T_{0}\right)$.

### 2.3. Thermal Energy and Momentum Equations

From Equations (12)–(14), the rate of change of specific internal energy can be partitioned using the *P* function and expressed as

(26) $$\begin{array}{c} \frac{De}{Dt}≃P\frac{De_{\ell }}{Dt}+\left(1-P\right)\frac{De_{s}}{Dt}+\left[L_{a}P^{\prime }+RT\chi^{\prime }G\left(\phi_{c}\right)\right]\frac{D\phi_{s\ell }}{Dt}+RT\chi G^{\prime }\left(\phi_{c}\right)\frac{D\phi_{c}}{Dt}, \end{array}$$

and with the Fourier law of conduction the thermal energy equation (Equation (6)) can be developed and written as

(27) $$\begin{array}{c} \rho c_{p}\frac{DT}{Dt}=\nabla \cdot (k_{T}\nabla T)-\rho \left[L_{a}P^{\prime }+RT\chi^{\prime }G\right]\frac{D\phi_{s\ell }}{Dt}-\rho RT\chi G^{\prime }\frac{D\phi_{c}}{Dt}-\dot{\Omega }, \end{array}$$

where $c_{p}$ is specific heat, $k_{T}$ is thermal conductivity, and both are defined as phase $\phi_{s\ell }$ and temperature dependent properties:

(28) $$c_{p}\left(T,\phi_{s\ell },\phi_{c}\right)={c_{p}}_{0}\tilde{c_{p}}≃{c_{p}}_{0}\left[P\left(\phi_{s\ell }\right){\tilde{c_{p}}}_{\ell }\left(T,\phi_{c}\right)+\left(1-P\right){\tilde{c_{p}}}_{s}\left(T\right)\right],$$

and

(29) $$k_{T}\left(T,\phi_{s\ell },\phi_{c}\right)={k_{T}}_{0}\tilde{k_{T}}≃{k_{T}}_{0}\left[P\left(\phi_{s\ell }\right){\tilde{k_{T}}}_{\ell }\left(T,\phi_{c}\right)+\left(1-P\right){\tilde{k_{T}}}_{s}\left(T\right)\right].$$

Therefore the interpolation *P* function defined previously is applied here for the transition of properties between solid and liquid phases. The corresponding reference values are defined as ${c_{p}}_{0}={c_{p}}_{\ell }\left(T\to T_{0}\right)$ and ${k_{T}}_{0}={k_{T}}_{\ell }\left(T\to T_{0}\right)$ based on pure water. The volumetric heat sink $\dot{\Omega }$ drives the overall freezing process for the simplified mesoscale model without considering actual heat transfer surfaces or wall conditions. Here we apply a linear cooling model:

(30) $$\dot{\Omega }=\rho c_{p}\beta_{f},$$

with a constant cooling rate $\beta_{f}$ to represent a uniform influence on the mesoscale domain based on cooling condition for the bulk environment. The parameter $\beta_{f}$ may be modified for multiscale simulation that couples the representative local model with a full scale CFD simulation at the vial or bottle level. In principle, directional cooling can also be applied to inspect the anisotropic growth of ice crystals.

The fluid flow within the liquid domain or in the confined interstitial space between ice crystals is primarily driven by the blowing mass flux due to density change upon freezing (or suction upon melting). The continuity equation based on local mass conservation is coupled with the phase field $\phi_{s\ell }$ and can be expressed as

(31) $$\nabla \cdot v=-\frac{1}{\rho }\frac{D\rho }{Dt}≃-\frac{1}{\rho }\frac{\partial \rho }{\partial \phi_{s\ell }}\frac{D\phi_{s\ell }}{Dt},$$

where v is velocity, ρ is density, and the material derivative D/Dt takes convective effect on the evolution of the phase field into account. The local density is defined as

(32) $$\rho \left(T,\phi_{s\ell },\phi_{c}\right)=\rho_{0}\tilde{\rho }≃\rho_{0}\left\{\phi_{c}\tilde{\rho_{c}}+\left(1-\phi_{c}\right)\left[P\left(\phi_{s\ell }\right)\tilde{\rho_{\ell }}\left(T,\phi_{c}\right)+\left(1-P\right)\tilde{\rho_{s}}\left(T\right)\right]\right\},$$

where the scaled density of ice crystals $\tilde{\rho_{s}}$ and supercooled water $\tilde{\rho_{\ell }}$ are temperature dependent, and $\rho_{0}=\rho_{\ell }\left(\phi_{c}\to 0,T\to T_{0}\right)$. The Navier-Stokes momentum equation with Boussinesq approximation for the buoyancy effect is given by

(33) $$\begin{array}{c} \rho \left(\frac{\partial v}{\partial t}+v\cdot \nabla v\right)=-\nabla p+\nabla \cdot \sigma_{v}+\left(\rho -\rho_{0}\right)g, \end{array}$$

where g is gravity acceleration, and $\sigma_{v}$ is the viscous stress for the assumed Newtonian fluid, written as

(34) $$\begin{array}{c} \sigma_{v}=\eta \left(\nabla v+\nabla v^{T}\right), \end{array}$$

where the dynamic viscosity η is defined as

(35) $$\eta \left(T,\phi_{s\ell },\phi_{c}\right)=\eta_{0}\tilde{\eta }≃\eta_{0}\left[P\left(\phi_{s\ell }\right){\tilde{\eta }}_{\ell }\left(T,\phi_{c}\right)+\left(1-P\right){\tilde{\eta }}_{s}\right],$$

with an assumed constant viscosity for the crystal phase ${\tilde{\eta }}_{s}\gg {\tilde{\eta }}_{\ell }$ and a reference value $\eta_{0}=\eta_{\ell }\left(\phi_{c}\to 0,T\to T_{0}\right)$. At the mesoscale, the buoyancy effect introduces a small updraft and shows tendency of collective motion of ice crystals. Furthermore, in the proposed phase-field model, instead of elastic solid material the ice crystals are treated as a highly viscous fluid with viscosity at least four orders of magnitude higher than liquid viscosity. To further simplify the computation, the fluid flow is assumed quasi-incompressible, so that the density variation is decoupled from pressure field except for the phase change interface and Boussinesq approximation. The modified pressure that incorporates the interfacial dynamics can be approximated by the pressure Poisson equation:

(36) $$\begin{array}{c} \nabla^{2}p≃\nabla \cdot \left[\nabla \cdot \sigma_{v}+\left(\rho -\rho_{0}\right)g-\rho v\cdot \nabla v\right]+\frac{\partial }{\partial t}\left(\frac{\partial \rho }{\partial \phi_{s\ell }}\frac{D\phi_{s\ell }}{Dt}\right), \end{array}$$

where the last term is considered an additional contribution to pressure due the non-solenoidal velocity field.

### 2.4. Scaling and Computation

The system has two length scales involved, the physical domain size 2πL and the apparent interfacial thickness $W_{s\ell }$ for computing phase transition. This yields six characteristic time scales based on solid-liquid phase transition, protein diffusion, thermal diffusion, viscous diffusion, convection, and the assumed freezing rate. These time scales are estimated by the following expressions:

(37) $$\begin{array}{c} \tau_{s\ell }=\frac{1}{\rho_{0}h_{s\ell }M_{s\ell }},\;\tau_{\phi_{c}}=\frac{L^{2}}{D_{0}},\;\tau_{{}_{T}}=\frac{\rho_{0}{c_{p}}_{0}L^{2}}{{k_{T}}_{0}},\\ \tau_{\mathrm{vis}}=\frac{\rho_{0}L^{2}}{\eta_{0}},\;\tau_{\mathrm{conv}}=\frac{L}{U}∼\tau_{\phi_{c}}∼\tau_{s\ell },\;\mathrm{and}\;\tau_{f}=\frac{▵T}{\beta_{f}}, \end{array}$$

respectively, where *U* is the characteristic velocity and ▵T represents the characteristic subcooled temperature. All governing equations are scaled by length *L* and phase transition time $\tau_{s\ell }$. The phase field $\phi_{s\ell }$, protein volume fraction $\phi_{c}$, χ parameter, *G* and *P* functions are already normalized. Temperature is scaled by the subcooled temperature as

(38) $$\begin{array}{c} \tilde{T}=\left(T-T_{0}\right)/▵T, \end{array}$$

and pressure and stress are scaled by the viscous effect based on the reference viscosity. From length scale *L* and time scale $\tau_{s\ell }$, the resulting scaled phase-field equations reduce to

(39) $$\begin{array}{cc} \frac{\partial \phi_{s\ell }}{\partial \tilde{t}}+P_{e}\tilde{v}\cdot & \tilde{\nabla }\phi_{s\ell }=C_{h}^{2}{\tilde{\nabla }}^{2}\phi_{s\ell }+\frac{\Lambda_{s\ell }\left(1-\phi_{c}\right)P^{\prime }\left(\tilde{T}-{\tilde{T}}_{eq}\right)}{\left(1+\frac{▵T}{T_{0}}\;\tilde{T}\right)\left(1+\frac{▵T}{T_{0}}\;{\tilde{T}}_{eq}\right)}\\ & +\left(1-\phi_{c}\right)\left(\phi_{s\ell }-\phi_{s\ell }^{3}\right)-\frac{R}{h_{s\ell }}\chi^{\prime }G, \end{array}$$

and

(40) $$\begin{array}{cc} \frac{\partial \phi_{c}}{\partial \tilde{t}}+P_{e} & \tilde{v}\cdot \tilde{\nabla }\phi_{c}=\frac{\tau_{s\ell }}{\tau_{\phi_{c}}}\tilde{\nabla }\cdot \{\tilde{D}\left[\frac{1-\phi_{c}}{N}+\phi_{c}+\left(\phi_{c}-\phi_{c}^{2}\right)\chi G^{\prime \prime }\right]\tilde{\nabla }\phi_{c}\\ & +\tilde{D}\left(\phi_{c}-\phi_{c}^{2}\right)G^{\prime }\tilde{\nabla }\chi \}-\frac{\tau_{s\ell }}{\tau_{\phi_{c}}}C_{h}^{2}\frac{h_{s\ell }}{R}\tilde{\nabla }\cdot \left\{\tilde{D}\left(\phi_{c}-\phi_{c}^{2}\right)\tilde{\nabla }{\tilde{\nabla }}^{2}\phi_{c}\right\}, \end{array}$$

where the Peclet number $P_{e}$, phase-change number $\Lambda_{s\ell }$, and the Cahn–Hilliard number $C_{h}$ are defined as

(41) $$\begin{array}{c} P_{e}=\frac{\tau_{\phi_{c}}\;\mathrm{or}\;\tau_{s\ell }}{\tau_{\mathrm{conv}}},\;\Lambda_{s\ell }=\frac{L_{a}▵T}{h_{s\ell }T_{0}^{2}},\;\mathrm{and}\;C_{h}=\frac{W_{s\ell }}{L}. \end{array}$$

Specifically the Peclet number measures the convective to diffusive effects on the redistribution of proteins, the phase-change number is the ratio of latent heat and interfacial energy, and the Cahn–Hilliard number controls the characteristic interfacial thickness to the length scale.

Furthermore, the scaled thermal energy equation can be expressed as

(42) $$\begin{array}{cc} \frac{\partial \tilde{T}}{\partial \tilde{t}}+ & P_{e}\tilde{v}\cdot \tilde{\nabla }\tilde{T}=L_{e}\tilde{\nabla }\cdot \left(\frac{\tilde{k_{T}}}{\tilde{\rho }\tilde{c_{p}}}\tilde{\nabla }\tilde{T}\right)\\ & -\frac{1}{\tilde{c_{p}}S_{\mathrm{te}1}}\left[P^{\prime }\left(\phi_{s\ell }\right)+S_{\mathrm{te}2}\left(\tilde{T}+\frac{T_{0}}{▵T}\right)\chi^{\prime }G\right]\left(\frac{\partial \phi_{s\ell }}{\partial \tilde{t}}+P_{e}\tilde{v}\cdot \tilde{\nabla }\phi_{s\ell }\right)\\ & -\frac{S_{\mathrm{te}2}}{\tilde{c_{p}}S_{\mathrm{te}1}}\left(\tilde{T}+\frac{T_{0}}{▵T}\right)\chi G^{\prime }\left(\frac{\partial \phi_{c}}{\partial \tilde{t}}+P_{e}\tilde{v}\cdot \tilde{\nabla }\phi_{c}\right)-\tilde{\dot{\Omega }}, \end{array}$$

where Peclet number $P_{e}$ has been defined above, interfacial Lewis number $L_{e}$ measures the ratio of interfacial evolution to thermal diffusion time scales, two Stefan numbers $S_{\mathrm{te}1}$ and $S_{\mathrm{te}2}$ compare the sensible heat and partition effect to the latent heat, respectively, and $\tilde{\dot{\Omega }}$ is the scaled cooling rate, defined as

(43) $$\begin{array}{c} \;L_{e}=\frac{\tau_{s\ell }}{\tau_{T}},\;S_{\mathrm{te}1}=\frac{{c_{p}}_{0}▵T}{L_{a}},\;S_{\mathrm{te}2}=\frac{R▵T}{L_{a}},\;\mathrm{and}\;\tilde{\dot{\Omega }}=\frac{\tau_{s\ell }}{\tau_{f}}, \end{array}$$

respectively. The scaled continuity equation is

(44) $$\begin{array}{c} \tilde{\nabla }\cdot \tilde{v}≃-\frac{\;1\;}{P_{e}\tilde{\rho }}\frac{\partial \tilde{\rho }}{\partial \phi_{s\ell }}\left(\frac{\partial \phi_{s\ell }}{\partial \tilde{t}}+P_{e}\tilde{v}\cdot \tilde{\nabla }\phi_{s\ell }\right). \end{array}$$

The scaled Navier-Stokes momentum equation can be expressed as

(45) $$\begin{array}{cc} \frac{1}{S_{c}}\frac{\partial \tilde{v}}{\partial \tilde{t}}+ & R_{e}\tilde{v}\cdot \tilde{\nabla }\tilde{v}=-\tilde{\nabla }\tilde{p}+\tilde{\nabla }\cdot \left[\tilde{\eta }\left(\tilde{\nabla v}+\tilde{\nabla }{\tilde{v}}^{T}\right)\right]-G_{r}{\hat{e}}_{g}, \end{array}$$

where ${\hat{e}}_{g}$ indicates the downward direction of gravity acceleration. The Schmidt number $S_{c}$ compares the phase transition to viscous time scales, Reynolds number $R_{e}$ characterizes the inertial to viscous effects and the local Grashof number $G_{r}$ measures the buoyancy to viscous forces adjusted by the local density, defined as

(46) $$\begin{array}{c} \;S_{c}=\frac{\tau_{s\ell }}{\tau_{\mathrm{vis}}},\;R_{e}=\frac{\rho_{0}UL}{\eta_{0}}=\frac{\tau_{\mathrm{vis}}}{\tau_{\mathrm{conv}}},\;\mathrm{and}\;G_{r}=\frac{\rho_{0}L^{2}g\left(1-\tilde{\rho }\right)}{\eta_{0}U}, \end{array}$$

respectively. The pressure can be obtained from the Poission equation (Equation (36)) and because of the small Reynolds number, the nonlinear inertial effect can be neglected.

In summary, the phase-field equations describe the transient evolution of the freezing front and redistribution of solutes in the liquid solution, the thermal energy equation determines the transient temperature distribution, and the continuity, momentum, and auxiliary pressure Poisson equations govern the interplay of fluid flow with phase fields and thermal energy. These equations are discretized and computed by our in-house Matlab codes with algorithms developed based on Fourier spectral method [38]. The two-dimensional computational domain includes 1600 × 1600 uniform collocation points and periodic boundary conditions. The built-in Matlab functions of fast Fourier and inverse Fourier transforms are applied in the computation. The temporal discretization is based on forward Euler scheme with a uniform and scaled time step 2 × $10^{-4}$ throughout the transient process. A pseudo spectral scheme is applied to the nonlinear terms of the governing equations. As the phase-field method naturally resolves deformation and coalescence of the crystals, there is no inter/extrapolation or any smooth or adaptive schemes applied to the interface or spatial derivatives in the computation. The initial conditions are based on predetermined nucleation sites and seeding size, and a uniform supercooled temperature for the onset of crystaliztion. The model is not limited to this simplified initial condition and can be further developed to incorporate various nucleation models. A semi-implicit scheme is applied to spatial or spectral domain for variable transport coefficients primarily associated with the 2nd-order derivatives [39]. The two-dimensional simulation results for the cases presented here are manageable by a conventional desktop computer with 16 gb ram, and for each case the transient simulation can be completed overnight.

## 3. Material Properties

First, water expands upon freezing and due to a smaller mass density of ice crystals than liquid solution, mass flux is generated at the freezing front toward the liquid side. We describe this effect by incorporating the temperature-dependent densities for the supercooled water and ice crystals based on the compiled dataset from the CRC Handbook [40], and formulated in terms of dimensional values in MKS (meter, kilogram, second) units as

(47) $$\begin{array}{c} \rho_{\ell }\left(T\right)≃\rho_{0}-0.017{\left(T-T_{0}\right)}^{2},\;\mathrm{and}\;\rho_{s}\left(T\right)≃0.917\rho_{0}+0.15\left(T_{0}-T\right). \end{array}$$

The sucrose density $\rho_{c}$ is assumed a constant 1587 kg/m^3^.

Second, the specific heat of ice crystals is assumed independent of solute volume fraction due to the strong exclusion effect, and decreases as temperature decreases, with data [40] correlated as

(48) $$\begin{array}{c} {c_{p}}_{s}\left(T\right)≃{c_{p}}_{0}+8\left(T-T_{0}\right). \end{array}$$

The specific heat of supercooled sucrose solution [8] is given by

(49) $$\begin{array}{c} {c_{p}}_{\ell }\left(T,\phi_{c}\right)≃4180\left[1-0.953\phi_{c}\left(1-0.588\phi_{c}\right)+10^{-3}\left(T-T_{0}\right)\right]. \end{array}$$

Third, the thermal conductivity of ice crystals increases as temperature decreases [40], approximated by

(50) $$\begin{array}{c} {k_{T}}_{s}\left(T\right)≃{k_{T}}_{0}+0.013\left(T_{0}-T\right). \end{array}$$

The thermal conductivity of supercooled sucrose solution [8] is approximated by

(51) $$\begin{array}{c} {k_{T}}_{\ell }\left(T,\phi_{c}\right)≃0.58\left[1-0.905\phi_{c}\left(1-0.588\phi_{c}\right)+2.6\times 10^{-3}\left(T-T_{0}\right)\right]. \end{array}$$

Several reference properties at temperature $T_{0}$ are denoted by the subscript 0 with test values listed in Table 1.

Fourth, the dynamic viscosity of water increases when temperature decreases. The temperature-dependent viscosity for the supercooled water can be correlated by the Vogel-Fulcher-Tamman (VTF) model [41]:

(52) $$\begin{array}{c} \eta_{\ell }\left(T\right)≃4.442\times 10^{-5}\;\exp\left(\frac{2.288\times 168.9}{T-168.9}\right), \end{array}$$

in which the prefactor $4.442\times 10^{-5}$ is the viscosity at temperature 168.9 K (>$T_{g}≃136\;K$). Note that the scaling factor for viscosity $\eta_{0}$ (∼0.0018 Pa·s) is defined at the freezing temperature $T_{0}$. At higher solute concentration, the mobility or self diffusivity of solutes can be significantly reduced due to molecular crowding effect and protein-protein interactions. Empirical model for the concentration-dependent diffusivity in the supercooled regime is required for a specific protein of interest. However, due to the lack of such data in the supercooled regime, here we compose the VTF model and the concentration-dependent viscosity of sucrose solutions measured at $T_{0}=273.15\;K$ [42]. With a conversion from weight percentage to volume fraction, the viscosity is correlated as

(53) $$\begin{array}{c} \eta_{\ell }\left(T,\phi_{c}\right)=\eta_{\ell }\left(T_{0},\phi_{c}\right)\eta_{\ell }\left(T\right)≃\exp\left(\frac{6.3\phi_{c}}{1-0.85\phi_{c}}\right)\eta_{\ell }\left(T\right), \end{array}$$

where the exponential term follows the Mooney’s viscosity model. In the crystal domain we assume $\eta_{s}≃10^{4}\eta_{0}$.

Finally, the reference solute diffusivity $D_{0}$ in sucrose solution at $T_{0}$ is about $2.1\times 10^{-10}$ [19], from which the temperature and concentration dependency can be estimated by the proportionality to temperature and viscosity based on the Stokes–Einstein relation and described as

(54) $$\begin{array}{c} D_{\ell }\left(T,\phi_{c}\right)≃\frac{D_{0}\;T\;\eta_{0}}{\;\;T_{0}\;\eta_{\ell }\left(T,\phi_{c}\right)\;}. \end{array}$$

In the crystal domain we assume $D_{s}≃10^{-4}D_{0}$. The liquidus temperature that accommodates the freezing-point depression effect is correlated from the sucrose-water phase diagram [43] as

(55) $$\begin{array}{c} T_{eq}\left(\phi_{c}\right)≃T_{0}-55\phi_{c}^{2}. \end{array}$$

A summary of the parameters and dimensionless groups used for the test cases are listed in Table 1 and Table 2.

## 4. Results and Discussion

**Figure 2:**
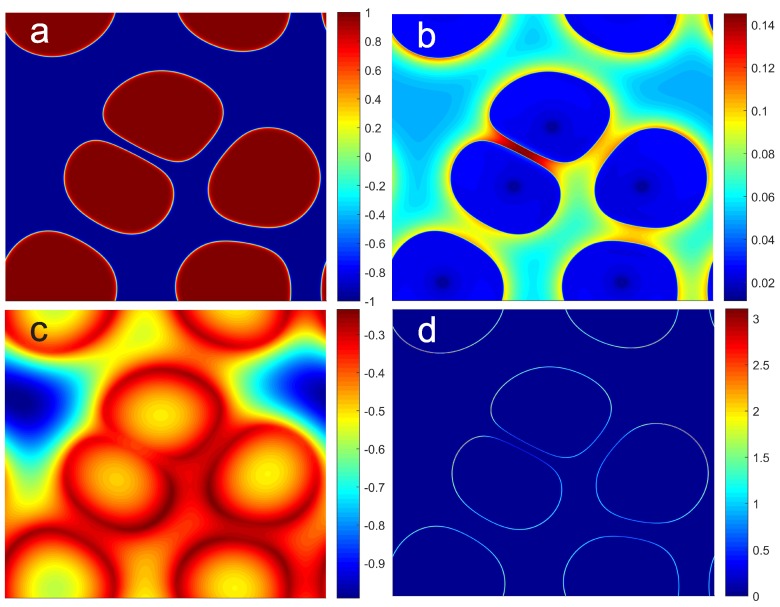
Snapshots of the scaled simulation results at time instant $\tilde{t}=1.5$, showing (**a**) phase field $\phi_{s\ell }$ that distinguishes the ice crystals (red) and liquid mixture (blue), (**b**) solute volume fraction $\phi_{c}$ due to freeze concentration, (**c**) temperature distribution $\tilde{T}$ that shows the exothermic effect, and (**d**) positive (freezing, or negative upon melting) volumetric flow rate $\tilde{\nabla }\cdot \tilde{v}$ induced at the ice/freeze concentrate interface due to density variation. Initial conditions are $\tilde{T}=-1.0$ (or dimensional temperature $T_{0}-10$ K), $\phi_{c}=0.05$ (about 7.7 wt %), and a rapid cooling rate with $\beta_{f}=100\;K/s$ or volumetric heat sink $\dot{\Omega }$ = $\rho c_{p}\beta_{f}≃$ 2 × 10^8^ J/m^3^·s, and the simulation has time scale $\tau_{s\ell }=$ 0.4762 s, length scale 10 μm, and domain size about 63×63 μm.

Figure 2 demonstrates the computational results at a scaled time instant $\tilde{t}=1.5$, showing the fully coupled thermal fluid and phase transition dynamics driven by a supercooled temperature $\tilde{T}=-1.0$ initially, and then by a uniform volumetric cooling rate with $\beta_{f}=100\;K/s$. The initial volume fraction is set to $\phi_{c}=0.05$ in the liquid mixture and $\phi_{c}=0$ for the seeding crystals. The phase field $\phi_{s\ell }$ (Figure 2a) has a sharp interface that clearly distinguishes the crystal domains from the liquid mixture. The solute volume fraction $\phi_{c}$ (Figure 2b) shows that solutes are pushed away by the interfaces as they grow into the liquid mixture. This is due to solute exclusion from the crystals, and the solute concentration is further enhanced between close-approached crystals as the space is confined. The small dark-blue spot near the center of each crystal implies the seeding site where $\phi_{c}=0$ initially. The narrow gap between ice/freeze concentrate interfaces further hinders diffusive mass transport with lower mass diffusivity due to the increase of viscosity at higher solute concentration. This is an additional influence on mass transfer due to temperature adjustment. As crystals approaches each other, the moving interfaces slow down and flatten, which creates a narrow gap in between. The slow down is due to higher local temperature from the exothermic effect upon freezing and the freezing point depression as local concentration increases. The solutes are mostly trapped in the narrow channels and the Plateau border areas between the interfaces. The temperature variation primarily due to heat conduction is shown in Figure 2c and the distribution is relatively diffuse compared with the phase and concentration fields. Near the freezing front the latent heat is released and this exothermic effect increases the local temperature significantly. Although thermal diffusion is much faster than mass transfer and phase transition, temperature variation over the computational domain is significant and thus an isothermal assumption often applied in solidification process simulation is not suitable here. The thermal convection effect is less important in the mesoscale regime due to relatively low Reynolds number and small Peclet and Grashof numbers. Because of smaller mass density of crystal phase than liquid solution, the volumetric flow rate (Figure 2d) creates a blowing mass flux from the ice/freeze concentrate interface towards the liquid solution. The local flux slightly enhances the mass transfer and the removal of latent heat.

**Figure 3:**
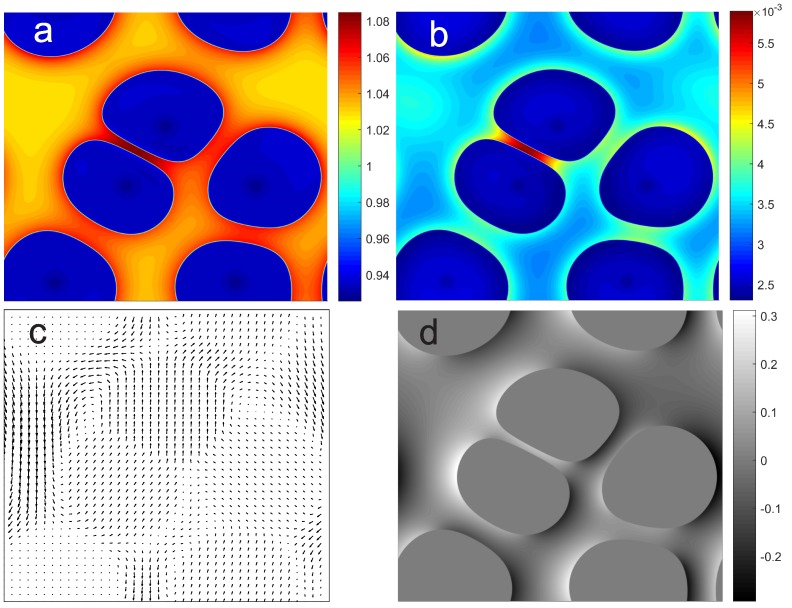
Computational results in addition to Figure 2 at time instant $\tilde{t}=1.5$: (**a**) local density $\tilde{\rho }$ adjusted by temperature and solute concentration, (**b**) dimensional liquid viscosity $\mu_{\ell }$ (unit Pa s, a dimensional value here for convenience), which is raised by higher solute concentration and lower supercooled temperature, (**c**) scaled velocity $\tilde{v}$ for the fluid flow driven by interfacial blowing and the gravity effect, and (**d**) scaled vorticity field corresponding to the velocity field.

Furthermore, the scaled density (Figure 3a) and liquid viscosity (Figure 3b) corresponding to Figure 2 have taken local temperature and solute volume fraction into account. This is important for describing reduced mobility of solutes at higher concentration and lower temperature. Again the interfaces can be slowed down significantly due to higher solution viscosity, freezing point depression, and higher local temperature due to the release of latent heat. Although the increase of temperature slightly reduces the viscosity and enhances the mass diffusivity, the higher temperature lowers the driving force for the phase transition significantly. From the velocity field (Figure 3c) one can observe a tendency of collective upward motion of crystals. This is due to buoyant effect in addition to the local mass flux. Finally, the vorticity field (Figure 3d) shows stronger circulation around the interstitial liquid space, which correlates well with the phase transition and higher temperature region.

**Figure 4:**
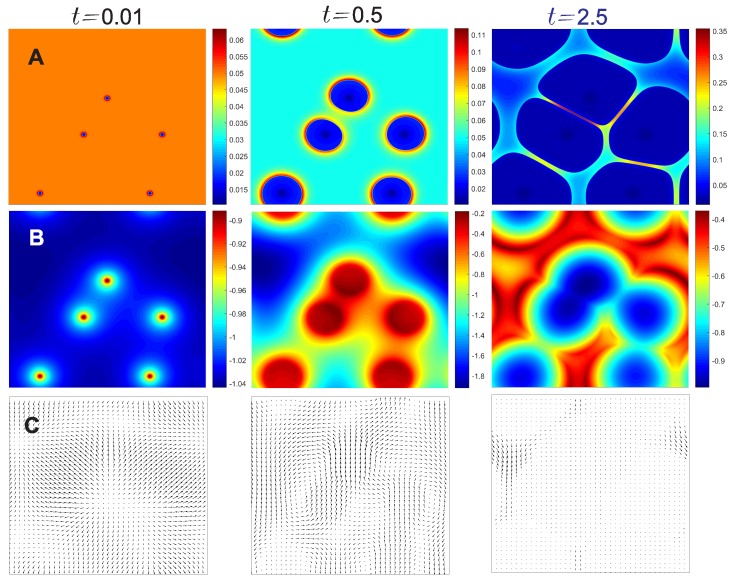
Transient results of solute volume fraction (**A**, 1st row), temperature (**B**, 2nd row), and scaled velocity vectors (**C**, 3rd row) at the corresponding time instants $\tilde{t}=0.01,0.5,\;\mathrm{and}\;2.5$. Parameters and initial conditions are the same as in Figure 2 and Figure 3, where the results at $\tilde{t}=1.5$ have been provided.

Figure 4 shows transient evolution of crystal-solution interfaces and freeze concentration between these crystals. The total number and locations of nuclei and the onset temperature ($\tilde{T}=-1.0$ or dimensional temperature $T=T_{0}-10$ K) of crystallization are predefined, same as in Figure 2 and Figure 3. The dynamic space confinement and freeze concentration are clearly resolved along with the fluid flow surrounding crystals, and for the movement of the crystals. In principle, as cooling continues, the non-equilibrium process may lead to fusion or coalescence of crystals. Solute exclusion from the crystal phase is controlled by the Flory’s interaction parameter χ, which can be an empirical property from experimental analysis. The topological structure of the growth of crystals is quite similar to those found in bubble or foam materials. The drainage of the fluid within the thin film and the Plateau border area is however not as significant as in foam materials, in which both the continuous and dispersed phases have fluid-like behaviors. At $\tilde{t}=0.5$, the scaled temperature spans a wide range from −1.8 to −0.2, this is because of the rapid cooling that competes with the release of latent heat. The raised temperature between two nearby crystals is clearly observed, with decelerated phase transition. At $\tilde{t}=2.5$, highest local concentration increases about seven-fold within the narrow gap between crystals, with maximum $\phi_{c}$ about 0.35. In principle, the concentration could further increase as cooling continues till $T_{g}^{\prime }$, but the relevant physics involving strong protein-protein interactions are beyond the scope of this model.

**Figure 5:**
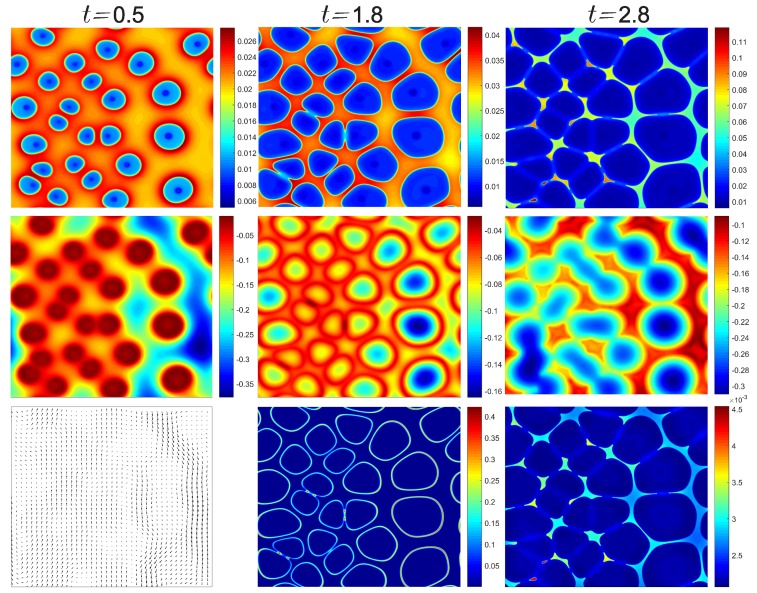
Transient results of solute volume fraction (1st row), temperature (2nd row), and velocity vectors, volumetric flow rate, and liquid viscosity on the 3rd row at time instants $\tilde{t}=0.5,1.8,\;\mathrm{and}\;2.8$, respectively. Initial conditions $\tilde{T}=-1.0$ and $\phi_{c}=0.02$ (about 3.1 wt %), and an assumed cooling rate with $\beta_{f}=100\;K/s$.

In Figure 5 we demonstrate a case with five times more nucleation sites (25 seeds in the computational domain) and lower initial protein volume fraction ($\phi_{c}=0.02$), but under the same cooling condition and onset temperature of crystallization. By arranging higher seeding density presumable on the left-hand side of the domain and with the same seeding size, the growth of crystals is relatively uneven as expected. At the early stage $\tilde{t}=0.5$ we observe a greatly increased temperature because of the larger amount of phase transition that happens simultaneously so that the released latent heat can not be removed efficiently. On the 3rd row, as many crystals grow spontaneously the collective updraft appears due to the buoyant effect, which also induces a locally sinking flow on the right-hand side of the domain that has more liquid solution and less crystals. At the later stage $\tilde{t}=2.8$, although freeze concentration in the interstitial space would suppress freezing, many crystals still coalesce with surrounding crystals. This creates several spots that have almost sixfold increase of solute volume fraction. Coalescence creates spotty areas with solutes trapped inside. Overall, the crystal phase grows faster around high-density nucleation sites, but such growth is limited by the large amount of latent heat released, whereas the loosely seeding site has more room for the crystals to grow in the later stage, showing an interesting growth pattern, solute composition, temperature map, and the surrounding fluid flows. All these effects, along with the initial conditions determine the growth of ice crystals and the degree of freeze concentration. We observe that the onset temperature of crystallization is relatively less important because even the initial temperature is near the homogeneous nucleation temperature about −40 °C, the amount of sensible heat $c_{p}\Delta T$ is about an order of magnitude smaller than the latent heat to be released. Therefore, without rapid cooling the sensible heat itself is insufficient to grow the crystals. The onset temperature may have influence on the sporadic precipitation or growth of dendritic microstructure, which will be investigated in future works.

In summary, freezing is a complicated exothermic process and the design and control of the process has a large parameter space. This preliminary and qualitative investigation has demonstrated a few important mechanisms involved in the freezing process. Dynamic freeze concentration due to volume exclusion and space confinement effect is demonstrated for a mesoscale system with domain size about tens of microns. Within the interstitial space between ice crystals, the increase of solute concentration suppresses the freezing point, reduces the solute mobility, and hinders the coalescence or further growth of ice crystals. Parameter and sensitivity tests with experimental validation are important for future investigation so that a lab-scale model can be further developed. The theoretical framework for a binary solution can be further extended to multiple components including co-solutes, and for a system under directional cooling or cooling from multiple heat transfer surfaces. Multiscale modeling, precision measurement of the transport properties especially in the supercooled regime, and the quantitative analysis of process dynamics for a larger scale experiment with configuration such as a vial, bag, or bottle that has volume from a few milliliters to tens of liters are all important for industrial applications. Finally the dimensionless groups list in Table 1 can be a reference for the design-of-experiment (DoE) approach for developing operation parameters or new manufacturing strategies to mitigate freeze concentration and enhance the quality of biologic products.

## 5. Conclusions

Freeze concentration is an important aspect of the freezing process in manufacturing of biopharmaceutical products. Freeze concentration alters the solution composition in which pharmaceutical proteins have been stabilized, often destabilizing the proteins. The freeze concentration affects ice crystal growth, which can also influence protein stability. We initiated a theoretical analysis on the dynamic evolution of ice crystals, crystal-crystal interactions, and the interplay of thermal, mass, and momentum transport phenomena with the dynamics of freeze concentration. The model is based on an asymmetric binary solution with thermophysical properties in the supercooled regime. A priori specified nucleation sites and a global and rapid cooling are applied to the computational domain. We found that the phase transition and freeze-concentration effect are largely controlled by the pattern of initial seeding and thermal transport. Other competing effects including mass diffusion, density variation, interstitial fluid flows, and the significantly increased local viscosity also play roles at different stages of the freezing process. The phase-field approach provides a great opportunity to model and investigate these process details.

## Figures and Tables

**Table 1:** Parameters, characteristic time scales, and reference properties for ice crystal and supercooled water used in the test cases. Parameter *N* is approximated by sucrose in water solution.

Parameters/Characteristic Times/Reference Properties	Value, SI
length scale *L*	$10^{-5}$ m
computational domain 2πL×2πL
interfacial thickness $W_{s\ell }$	$10^{-6}$ m
reference freezing temperature $T_{0}$	273.15 K
characteristic subcooled temperature ▵T	10 K
energy barrier coefficient $h_{s\ell }$	0.1456 J/(kg·K)
Flory’s parameters $\chi_{s}$, $\chi_{\ell }$	2.5, 0.5
solute-to-water ratio of molar volume *N*	∼11.5
scaled time step *h*	$2\times 10^{-4}$
latent heat $L_{a}$	$3.4\times 10^{5}$ J/kg
interfacial energy $\gamma_{s\ell }$	0.0375 J/m^2^
cooling rate $\beta_{f}$	100 K/s
initial temperature $T\left(t=0\right)=T_{0}-▵T$	263.15 K
characteristic phase transition time $\tau_{s\ell }$	0.4762 s
mass diffusion time $\tau_{c}$	0.4762 s
convective time $\tau_{\mathrm{conv}}$	0.4762 s
freezing time $\tau_{f}$	0.1 s
thermal diffusion time $\tau_{T}$	$9.86\times 10^{-5}$ s
viscous diffusion time $\tau_{\mathrm{vis}}$	$9.86\times 10^{-5}$ s
density $\rho_{0}$	999.8 $\mathrm{kg}/m^{3}$
specific heat ${c_{p}}_{0}$	2110 J/(kg·K)
thermal conductivity ${k_{T}}_{0}$	2.14 W/(m·K)
solute diffusivity $D_{0}$	$2.1\times 10^{-10}\;m^{2}/s$
dynamic viscosity $\eta_{0}$	$1.8\times 10^{-3}\;\mathrm{Pa}\cdot s$

**Table 2:** Dimensionless groups with values based on parameters listed in Table 1.

Dimensionless Groups	Value
Peclet number $P_{e}$	1.0
Reynolds number $R_{e}$	$1.16\times 10^{-4}$
phase-change number $\Lambda_{s\ell }$	312.88
Cahn–Hilliard number $C_{h}$	0.1
interfacial Lewis number $L_{e}$	4830.6
Stefan number ${S_{\mathrm{te}}}_{1}$	0.0621
Stefan number ${S_{\mathrm{te}}}_{2}$	0.0136
uniform cooling rate $\tilde{\dot{\Omega }}$	4.762
Schmidt number $S_{c}$	8620.8
local Grashof number $G_{r}$	25.92 ($1-\tilde{\rho }$)

## References

[1] Bhatnagar B.S., Bogner R.H., Pikal M.J. (2007). Protein stability during freezing: Separation of stresses and mechanisms of protein stabilization. Pharm. Dev. Technol..

[2] Bhatnagar B.S., Pikal M.J., Bogner R.H. (2008). Study of the individual contributions of ice formation and freeze-concentration on isothermal stability of lactate dehydrogenase during freezing. J. Pharm. Sci..

[3] Kasper J.C., Friess W. (2011). The freezing step in lyophilization: Physico-chemical fundamentals, freezing methods and consequences on process performance and quality attributes of biopharmaceuticals. Eur. J. Pharm. Biopharm..

[4] Miller M.A., Rodrigues M.A., Glass M.A., Singh S.K., Johnston K.P., Maynard J.A. (2013). Frozen-state storage stability of a monoclonal antibody: Aggregation is impacted by freezing rate and solute distribution. J. Pharm. Sci..

[5] Rodrigues M.A., Miller M.A., Glass M.A., Singh S.K., Johnston K.P. (2011). Effect of freezing rate and dendritic ice formation on concentration profiles of proteins frozen in cylindrical vessels. J. Pharm. Sci..

[6] Singh S.K., Kolhe P., Mehta A.P., Chico S.C., Lary A.L., Huang M. (2011). Frozen state storage instability of a monoclonal antibody: Aggregation as a consequence of trehalose crystallization and protein unfolding. Pharm. Res..

[7] Kasper J.C., Pikal M.J., Friess W. (2013). Investigations on polyplex stability during the freezing step of lyophilization using controlled ice nucleation—The importance of residence time in the low-viscosity fluid state. J. Pharm. Sci..

[8] Rodrigues M.A., Balzan G., Rosa M., Gomes D., de Azevedo E.G., Singh S.K., Matos H.A., Geraldes V. (2013). The Importance of heat flow direction for reproducible and homogeneous freezing of bulk protein solutions. AIChE J..

[9] Kobayashi R. (1993). Modeling and numerical simulations of dendritic crystal growth. Physica D.

[10] Warren J.A., Boettinger W.J. (1995). Prediction of dendritic growth and microsegregation patterns in a binary alloy using the phase-field method. Acta Metall. Mater..

[11] Murray B.T., Wheeler A.A., Glicksman M.E. (1995). Simulations of experimentally observed dendritic growth behavior using a phase-field model. J. Cryst. Growth.

[12] Karma A., Rappel W.J. (1996). Phase-field method for computationally efficient modeling of solidification with arbitrary interface kinetics. Phys. Rev. E.

[13] Karma A., Rappel W.J. (1998). Quantitative phase-field modeling of dendritic growth in two and three dimensions. Phys. Rev. E.

[14] Beckermann C., Diepers H.-J., Steinbach I., Karma A., Tong X. (1999). Modeling melt convection in phase-field simulation of solidification. J. Comput. Phys..

[15] Boettinger W.J., Warren J.A., Beckermann C., Karma A. (2002). Phase-field simulation of solidification. Annu. Rev. Mater. Res..

[16] Nakagawa K., Hottot A., Vessot S., Andrieu J. (2007). Modeling of freezing step during freeze-drying of drugs in vials. AIChE J..

[17] Radmanovic N., Serno T., Joerg S., Germershaus O. (2013). Understanding the freezing of biopharmaceuticals: First-principle modeling of the process and evaluation of its effect on product quality. J. Pharm. Sci..

[18] Roessl U., Jajcevic D., Leitgeb S., Khinast J.G., Nidetzky B. (2013). Characterization of a laboratory-scale container for freezing protein solutions with detailed evaluation of a freezing process simulation. J. Pharm. Sci..

[19] Butler M.F. (2002). Freeze concentration of solutes at the ice/solution interface studied by optical interferometry. Cryst. Growth Des..

[20] Kaempfer T.U., Plapp M. (2009). Phase-field modeling of dry snow metamorphism. Phys. Rev. E.

[21] Van der Sman R.G.M. (2016). Phase field simulation of ice crystal growth in sugar solutions. Int. J. Heat Mass Transf..

[22] Huang T.-H., Huang T.-H., Lin Y.-S., Chang C.-H., Chen P.-Y., Chang S.-W., Chen C.-S. (2018). Phase-field modeling of microstructural evolution by freeze-casting. Adv. Eng. Mater..

[23] Li J.-Q., Fan T.-H., Taniguchi T., Zhang B. (2018). Phase-field modeling on laser melting of a metallic powder. Int. J. Heat Mass Transf..

[24] Li J.-Q., Fan T.-H. (2018). Phase-field modeling of metallic powder-substrate interaction in laser melting process. Int. J. Heat Mass Transf..

[25] Van der Waals J.D. (1979). The thermodynamics theory of capillarity under the hypothesis of a continuous variation of density. J. Stat. Phys..

[26] Cahn J.W., Hilliard J.E. (1958). Free energy of a nonuniform system. I. interfacial free energy. J. Chem. Phys..

[27] Hohenberg P.C., Halperin B.I. (1977). Theory of dynamic critical phenomena. Rev. Mod. Phys..

[28] Penrose O., Fife P.C. (1990). Thermodynamically consistent models of phase-field type for the kinetics of phase transitions. Physica D.

[29] Wang S.-L., Sekerka R.F., Wheeler A.A., Murray B.T., Coriell S.R., Braun R.J., McFadden G.B. (1993). Thermodynamically-consistent phase-field models for solidification. Physica D.

[30] Anderson D.M., McFadden G.B., Wheeler A.A. (1998). Diffuse-interface methods in fluid mechanics. Annu. Rev. Fluid Mech..

[31] Anderson D.M., McFadden G.B., Wheeler A.A. (2000). A phase-field model of solidification with convection. Physica D.

[32] Bi Z., Sekerka R.F. (1998). Phase-field model of solidification of a binary alloy. Physica A.

[33] Yue P., Feng J.J., Liu C., Shen J. (2004). A diffuse-interface method for simulating two-phase flows of complex fluids. J. Fluid Mech..

[34] Takae K., Onuki A. (2011). Phase-field model of solid-liquid phase transition with density difference and latent heat in velocity and elastic fields. Phys. Rev. E.

[35] Lamorgese A.G., Molin D., Mauri R. (2011). Phase field approach to multiphase flow modeling. Milan J. Math..

[36] Roos Y.H. (2010). Glass transition temperature and its relevance in food processing. Annu. Rev. Food Sci. Technol..

[37] Doi M. (2013). Soft Matter Physics.

[38] Canuto C., Hussaini M.Y., Quarteroni A., Zang T.A. (1993). Spectral Methods in Fluid Dynamics.

[39] Zhu J., Chen L.-Q., Shen J., Tikare V. (1999). Coarsening kinetics from a variable-mobility Cahn–Hilliard equation: Application of a semi-implicit Fourier spectral method. Phys. Rev. E.

[40] Lide D.R. (2005). CRC Handbook of Chemistry and Physics.

[41] Dehaoui A., Issenmann B., Caupin F. (2015). Viscosity of deeply supercooled water and its coupling to molecular diffusion. Proc. Natl. Acad. Sci. USA.

[42] Swindells J.F., Snyder C.F., Hardy B.C., Golden P.E. (1958). Viscosities of sucrose solutions at various temperatures: Tables of recalculated values. NBS Circ..

[43] Young F.E., Jones F.T. (1949). Sucrose Hydrates. The sucrose-water phase diagram. J. Phys. Chem..

